# Grey-matter correlates of empathy in 4-Repeat Tauopathies

**DOI:** 10.1038/s41531-023-00576-z

**Published:** 2023-09-27

**Authors:** Benedetta Tafuri, Daniele Urso, Salvatore Nigro, Luigi Macchitella, Roberto De Blasi, K. Ray Chaudhuri, Giancarlo Logroscino

**Affiliations:** 1 Center for Neurodegenerative Diseases and the Aging Brain, University of Bari ‘Aldo Moro’, “Pia Fondazione Cardinale G. Panico”, Tricase, Lecce, Italy; 2 Department of Translational Biomedicine and Neuroscience (DiBraiN), University of Bari ‘Aldo Moro’, Bari, Italy; 3 Department of Neurosciences, King’s College London, Institute of Psychiatry, Psychology and Neuroscience, De Crespigny Park, London, UK; 4 Institute of Nanotechnology (NANOTEC), National Research Council, Lecce, Italy; 5 IRCCS “E. Medea”- Unit for Severe disabilities in developmental age and young adults (Developmental Neurology and Neurorehabilitation), Brindisi, Italy

**Keywords:** Parkinson's disease, Cognitive ageing

## Abstract

Loss of empathy is an early and central symptom of frontotemporal lobar degeneration spectrum diseases. We aimed to investigate the topographical distribution of morphometric brain changes associated with empathy in Progressive Supranuclear Palsy (PSP) and Corticobasal Syndrome (CBS) patients. Twenty-seven participants with CBS and 31 with PSP were evaluated using Interpersonal Reactivity Index scales in correlation with gray matter atrophy using a voxel-based morphometry approach. Lower levels of empathy were associated with an increased atrophy in fronto-temporal cortical structures. At subcortical level, empathy scores were positively correlated with gray matter volume in the amygdala, hippocampus and the cerebellum. These findings allow to extend the traditional cortico-centric view of cognitive empathy to the cerebellar regions in patients with neurodegenerative disorders and suggest that the cerebellum may play a more prominent role in social cognition than previously appreciated.

## Introduction

Empathy describes the ability to understand and share other’s emotional feelings and is fundamental to our emotional and social lives^1^. The two main neurocognitive components of empathic abilities are cognitive and emotional empathy. Cognitive empathy refers to cognitive ability of understanding of another person’s perspective and feelings (i.e. “I understanding what you feel”). Emotional empathy concerns the ability to feel and share the emotional experience of others (i.e. “I feel what you feel”). The neuroanatomical bases of these abilities in humans have been most consistently identified in a circuit comprising the insula, prefrontal cortices, superior temporal gyrus, temporoparietal junction and temporal poles, anterior cingulate cortex, and the inferior parietal lobules^1–3^, which are also regions that have been linked to the mirror neuron system^4^. However, whether socio-cognitive skills (including empathy) are functions independent from other nonsocial cognitive functions (e.g. memory or executive functions) with their own specialized brain modules is an open question in the literature.

While imaging of healthy subjects is necessary to identify the brain circuits involved in empathy^5–7^, complementary data from patients with neurodegeneration can add information about the relative importance of particular structures in those circuits, and can demonstrate which structures are required for normal empathy in real-life situations and which are not^8^. Notably, empathy impairment may be present across different neurodegenerative diseases with neuroanatomical underpinnings of that may be different across different disorders^9,10^. Loss of empathy can be a manifestation of several neurodegenerative disorders and can be an early and central symptom of frontotemporal lobar degeneration (FTLD), a neurodegenerative disorder characterized by the selective degeneration of the frontal and temporal cortices^11,12^. Emerging evidence indicates that difficulties in empathy abilities may also characterize both Progressive Supranuclear Palsy (PSP)^13^ and Corticobasal Syndrome (CBS)^9,14^, which are considered part of the FTLD spectrum. Interestingly, Arshad et al.^15^ investigated the empathy across different FLTD spectrum diseases, including behavioral variant Frontotemporal Dementia (bvFTD), primary progressive aphasia (PPA), amyotrophic lateral sclerosis (ALS) and PSP, finding that among these different neurodegenerative diseases, PSP patients manifest the maximal impairment in empathy abilities. However, the clinical and neuroanatomical correlates of empathy in PSP and CBS are not fully established and have been explored by only a limited number of studies^9,13,16,17^. Of particular interest, Ghosh et al. (2012) explored the social cognitive deficits and neural correlates for PSP patients by examine understanding of emotions in the voice^13^. They found that impairment of vocal emotion recognition was associated with atrophy of inferior frontal and cerebellar regions. Another previous study analyzed the relative neural correlates over 123 FTD patients, but only included a very small sample of CBS/PSP subjects^18^.

The purpose of the present study is to investigate the brain correlates of empathy deficits in a relatively large sample of PSP and CBS patients using a voxel-based morphometry (VBM) approach. To determine the specificity of these findings, we conducted a comparative analysis of the neural correlates of empathy deficits in behavioral variant Frontotemporal Dementia (bvFTD). We also explored whether empathy in these disorders is associated with general cognitive functions. We hypothesized that empathy deficits in PSP and CBS are independent of general cognitive functions and correlate with atrophy in frontotemporal regions.

## Results

### Clinical variables

The demographic and clinical characteristics of the PSP,CBS and bvFTD patients are summarized in Table 1. Significant differences were found in age distribution comparing PSP patients with CBS and bvFTD patients. PSP patients had higher PSPRS scores compared to patients with CBS (*p* = 0.018) and bvFTD (*p* < 0.001). Additionally, UPDRS III was higher for PSP respect to bvFTD (*p* < 0.001). IRI total score and IRI subscores (IRI-EC and IRI-PT) were similar among the three groups of patients (*p* = 0.360 and *p* = 0.603, respectively). Correlations between IRI and motor and cognitive variables are reported in Supplementary Table 1. We found no significant associations between IRI total score, IRI-EC, and IRI-PT subscores with any motor scores or cognitive tests.

**Table 1:** Demographic and clinical features associated with PSP, CBS and bvFTD patients.

	PSP	CBS	bvFTD	*p*-values
n	31	27	15	
Age (mean (SD))	69.94 (7.91)	65.81 (6.63)	62.60 (6.20)	0.004^a^
Male (%)	14 (45.2)	12 (44.4)	9 (60.0)	0.576
Disease duration (mean (SD))	5.00 (3.49)	4.63 (2.19)[4]	NaN [15]	0.642
Education (mean (SD))	16.10 (4.03) [1]	17.00 (4.84)[1]	14.60 (2.32)	0.201
MMSE score (mean (SD))	25.29 (3.70) [2]	24.44 (5.41)	22.07 (5.22)	0.098
CDR score (mean (SD))	0.69 (0.59)	0.67 (0.52)	1.30 (0.53)	0.001^b^
CDR Sum Of Boxes (mean (SD))	3.63 (2.99)	3.35 (2.94)	7.03 (2.51)	<0.001^c^
PSPRS (mean (SD))	34.59 (14.95)[4]	25.70 (9.73)[4]	8.30 (5.60)[5]	<0.001^d^
UPDRS III (mean (SD))	31.70 (17.90)	34.44 (15.60)[1]	4.85 (5.13)[2]	<0.001
SEADL (mean (SD))	55.38 (28.46)[4]	59.57 (20.33)[5]	65.00 (20.14)[5]	0.556
FAQ (mean (SD))	13.79 (7.53)[1]	10.69 (7.89)[2]	17.93 (5.98)	0.013
GDS-15 scale (mean (SD))	6.35 (4.33)[5]	4.73 (3.79)	3.82 (3.19)[4]	0.135
Total IRI (mean (SD))	44.03 (11.70)	46.04 (12.38)	53.67 (50.80)	0.472
IRIEC (mean (SD))	24.10 (6.10)	25.74 (6.90)	29.73 (24.65)	0.360
IRIPT (mean (SD))	19.94 (6.46)	20.30 (6.56)	23.93 (26.41)	0.603

Data are presented as mean (Standard Deviation or percentage). Missing data are reported in square brackets.

^a^PSP > CBS, *p* = 0.03; PSP > bvFTD, *p* = 0.003.

^b^bvFTD > PSP, *p* = 0.001.

^c^bvFTD > PSP, *p* < 0.001.

^d^PSP > CBS, *p* = 0.01; PSP > bvFTD, *p* < 0.001.

### Voxel-based morphometry

**Fig. 1:**
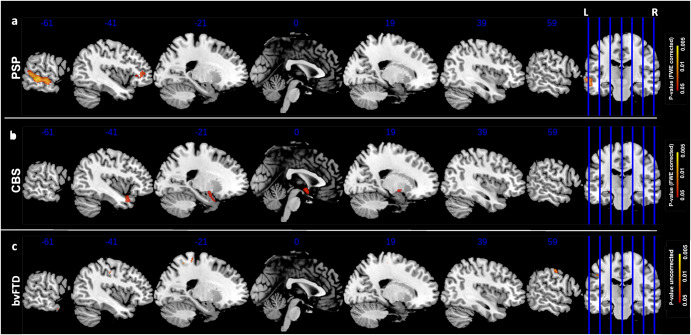
IRI total score correlations. Correlation p-maps between IRI total scores and smoothed GM of PSP (FWE-corrected, *p*-value < 0.05) (**a**), CBS (FWE-corrected, *p*-value < 0.05) (**b**) and bvFTD subjects (uncorrected *p*-value < 0.05) (**c**). The design matrix for this whole-brain voxel-based morphometry analysis contained IRI total score, with sex, age, TIV and CDR Sum of Boxes included as nuisance covariates. Abbreviations: IRI Interpersonal Reactivity Index, GM Gray Matter, PSP Progressive Supranuclear Palsy, CBS Corticobasal Syndrome, FWE Family Wise Error.

The associations between GM volume and IRI total score are reported in Fig. 1. Correlation analysis in PSP subjects, as reported in Fig. 1a, highlighted the left middle and superior temporal gyri as most correlated region (*p*-value < 0.05, FWE-corrected) with IRI total score, besides the left frontal orbital cortex. On the other hand, IRI total score in CBS subjects showed statistically significant correlations with GM volume in temporal pole bilaterally, also involving left hippocampus and amygdala(Fig. 1b, *p*-value < 0.05, FWE-corrected).

**Fig. 2:**
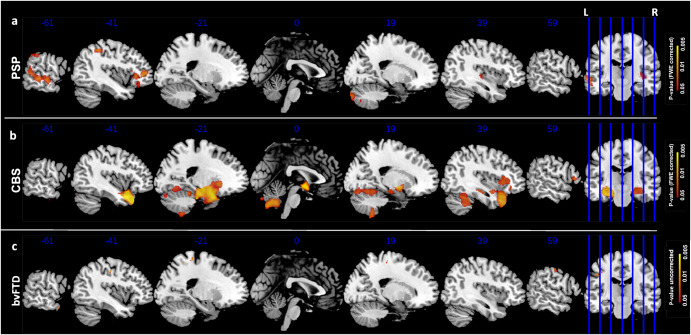
IRI-EC correlations. Correlation p-maps between EC subscores and smoothed GM of PSP (FWE-corrected, *p*-value < 0.05) (**a**), CBS (FWE-corrected, *p*-value < 0.05) (**b**) and bvFTD subjects (uncorrected *p*-value < 0.05) (**c**). The design matrix for this whole-brain voxel-based morphometry analysis contained only EC subscore, with sex, age, TIV and CDR Sum of Boxes included as nuisance covariates. Abbreviations: IRI Interpersonal Reactivity Index, EC Empathic Concern, GM Gray Matter, PSP Progressive Supranuclear Palsy, CBS Corticobasal Syndrome, FWE Family Wise Error.

**Fig. 3:**
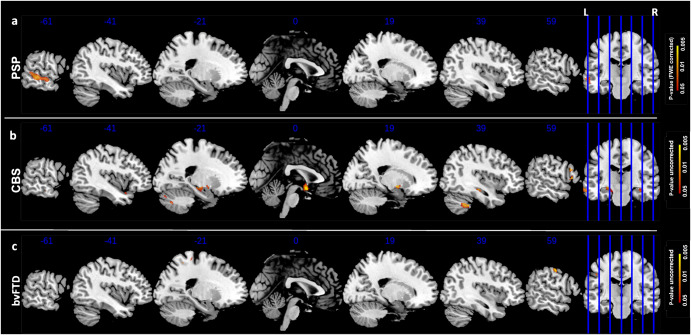
IRI-PT correlations. Correlation p-maps between PT subscores and smoothed GM of PSP (FWE-corrected, *p*-value < 0.05) (**a**), CBS (uncorrected *p*-value < 0.05) (**b**) and bvFTD subjects (uncorrected *p*-value < 0.05) (**c**). The design matrix for this whole-brain voxel-based morphometry analysis contained only PT subscore, with sex, age,TIV and CDR Sum of Boxes included as nuisance covariates. Abbreviations: IRI Interpersonal Reactivity Index, PT Perspective Taking, GM Gray Matter, PSP Progressive Supranuclear Palsy, CBS Corticobasal Syndrome, FWE Family Wise Error.

Similarly, Fig. 2 and Fig. 3 reported correlations between GM volume and EC and PT subscores, respectively. In particular, EC subscore (Fig. 2) showed similar patterns of correlation as for total IRI evaluation for all analyses. Notably, empathic concern in both PSP (Fig. 2a) and CBS (Fig. 2b) patients reported an additional statistically significant cluster of correlation in the cerebellum, involving predominantly Crus II. In CBS patients, we also found the involvement of subcortical regions, mostly of hippocampus and amygdala bilaterally. Finally, PT subscore correlation’s maps (Fig. 3) reported only one significant cluster survived for PSP subjects in left middle temporal gyrus (Fig. 3a, *p*-value < 0.05, FWE-corrected), while no significative correlation was found between PT subscore and CBS patients (Fig. 3b, *p*-value < 0.05, uncorrected).

To substantiate our finding, and to explore whether the neural correlates of empathy in PSP and CBS were different than in bvFTD, we examined brain correlates of empathy deficits in a sample of bvFTD subjects. As reported in Figs. 1c–2c–3c, no significant correlation was found between IRI scores and GM volume (only *p*-value < 0.05 uncorrected). However, sensitivity analysis discovered two main clusters in the right precentral and left postcentral gyri for the IRI total score and similarly for each subscore, with no involvement of subcortical regions. All information about clusters (e.g. number of voxels, coordinates) are reported in Supplementary Table 2.

## Discussion

In this study, VBM analysis was used to investigate correlations between gray matter volumes and measures of empathy in patients with PSP and CBS. We demonstrated that empathy deficits are independent from general cognitive functions in PSP and CBS. The primary finding was that lower levels of empathy were significantly associated with atrophy of fronto-temporal cortical structures, particularly the left superior and middle temporal gyrus and temporal pole bilaterally. We also found that atrophy in subcortical structures, and in particular the cerebellum may underlie empathy deficit in PSP and CBS patients. Additionally, we observed different neural correlates in patients with behavioral variant Frontotemporal Dementia (bvFTD), suggesting that those findings are specific to 4R-tauopathies.

We found that empathy scores correlated with volumes of several cortical areas, including the middle and superior temporal gyri, the inferior frontal gyrus and the temporal pole. The middle temporal gyrus is a core region of emotion generation and processing^19,20^, participating in processing cognitive empathy^21,22^ and theory of mind^23^. One study in healthy individuals found that, compared with the control stimuli, emotional exclamations of others’ pain elicited increased activation in the superior and middle temporal gyri, suggesting that these regions are involved in empathy^24^. Another study^5^, investigating the functional correlated of empathy using functional MRI (fMRI), found activations in the middle temporal gyrus and inferior frontal gyri when respondents had to make empathic judgments in a verbal task. More recently, Jie et al.^25^ showed that both empathy and counter-empathy, which refers to emotional reactions that are incongruent (or even at odds with the emotional states of others) shared a common neural mechanism in the middle temporal gyrus.

The superior temporal gyrus, which is connected to the limbic system via the insula, is another brain region that is important for numerous aspects of social cognition, and it has been shown to be involved in emotional empathy^26,27^. Evidence suggests that the superior temporal sulcus region is involved in social cognitive tasks, such as the passive perception of social scenes^28,29^, and in emotional content of the facial stimuli^30^. Interestingly, inferior temporal gyrus is involved in visual processing and is associated with the representation of objects, places, faces, and colors^31^. Moreover, a fMRI study showed that activity in a region of the posterior inferior temporal lobe was related to cognitive empathy^32^.

The role of temporal pole in social and emotional processing has been extensively explored in the literature. This region lies between the orbital frontal cortex and the amygdala and receives and sends connections to both regions, coupling emotional responses to highly processed sensory stimuli. One of the first hints that the temporal pole was involved in socioemotional processing came from studies of Klüver–Bucy syndrome^33^. Many functional neuroimaging studies^34,35^ found the temporal poles implied in theory of mind, as the ability to infer the desires, intentions, or beliefs of others. Vollm and colleagues^21^ also found that activation clusters for a theory of mind task and an empathy task overlapped in the temporal pole. Interestingly, a VBM study on empathy over FTLD spectrum found that the total empathy score, measured using the IRI score, the same measure of empathy that we have used in the current study, were correlated with structural MRI brain volume in the temporal pole and the fusiform gyrus^18^.

We have also found that subcortical structures, such as the amygdala, hippocampus and the cerebellum, correlates with empathy scores in patients with PSP and CBS. Previous studies have highlighted how volume loss in the amygdala and hippocampus may play a role in empathy deficits^36–38^. More interesting, and somewhat unexpected finding concerns the possible involvement of the cerebellum in empathic processes of these neurodegenerative disease. Previous studies on PSP and CBS patients had just found a possible involvement of cerebellar regions in ability to understand and share other’s emotions^14^. In particular for PSP, Ghosh et al.^13^ demonstrates a correlation between atrophy of the cerebellum and the task of understanding of emotions in the voice. However, the cerebellum has only recently been recognized as one of the major structures involved in empathy^16,39^. Meta-analyses of functional MRI studies of emotional empathy, particularly for other people’s feelings of pain, have shown activation of cerebellum in association with the emotional empathy task^40^. The cerebellar involvement in cognitive empathy fits with the significant covariation of the cerebellum with self-rated individual differences in empathy for pain described by Singer and colleagues^40^, and with the reported cerebellar involvement in social cognition^41,42^. On the other hand, mounting evidence suggests an association between cerebellar atrophy and cognitive impairment in the main frontotemporal dementia syndromes. A recent study^43^ aimed to comprehensively chart profiles of cognitive impairment in relation to cerebellar atrophy in 49 demented patients with PSP and CBS, revealing distinct cerebellar subregions differentially implicated across cognitive domains in each patient group and offering insights into the cerebellum’s contribution to cognitive processing in these neurodegenerative disorders. Furthermore, an association between alterations in cerebellum and deficit in empathy and in theory of mind have been showed in behavioural variant FTD and AD^44,45^. Overall, our findings indicate that the cerebellum may be of one of the main brain regions involved in empathic processes in both PSP and CBS highlighting its involvement in socio-cognitive processes (the “social cerebellum”^39^). This is also in line with the concept that a damage of the cerebellum can cause the cerebellar cognitive affective syndrome (CCAS), also called Schmahmann’s syndrome, which is characterized by deficits in executive function, linguistic processing, spatial cognition, affect and social regulation^46^.

In this study we also investigated the possible association of non-social cognitive functions with the empathic capabilities. Ghosh et al.^13^ stressed that the deficit of theory of mind in PSP appear not to be affected by concomitant deficits in executive function. Here, we assess the correlations between IRI and performance in wide range of neuropsychological tasks of non-social cognitive functions. We did not find any correlation between empathy and other non-social cognitive skills (i.e. memory, executive function). This evidence suggests that empathy in PSP and CBS may be independent by other non-social cognitive functions.

There are some limitations and future directions to be considered. First, understanding of the precise structural contributions to empathic dysfunction in both CBS and PSP would require the inclusion of more subjects and multimodal MRI approaches that measures of structural and functional connectivity between brains regions. Secondly, Alzheimer’s Disease (AD) is found to be the primary underlying pathology in ~25% of patients presenting with CBS^47^. In this study, “in vivo” biomarkers of AD and neuropathology were unavailable, therefore we could not identify CBS patients with underlying AD. Future neuroimaging studies assessing empathy should be performed in CBS patients with AD and non-AD pathology and should include cohorts of patient’s with typical AD and healthy controls to further confirm the specificity of neural correlates of empathy. Furthermore, a variety of measures have been developed in order to quantify empathy in neurodegenerative disorders, such as Multifaceted Empathy Test (MET) or Empathy Quotient (EQ)^48^. In this study we used the Interpersonal Reactivity Index as the most common questionnaire to quantify empathy. However, our results may be influenced by reporter bias and must be replicated through the use of different types of empathy measurement.

In conclusion, we sought to characterize empathy deficit and their neuroanatomic correlates in both PSP and CBS. We found that empathy score was independent of general cognitive functions and that correlated with of several cortical areas, including the middle temporal gyrus, inferior temporal gyrus, the temporal pole, the fusiform and lingual gyrus. We also found that atrophy in subcortical structures, and in particular the cerebellum, may underlie empathy deficit in PSP and CBS patients. These findings allow to extend the traditional cortico-centric view of cognitive empathy to the cerebellar regions in patients with neurodegenerative disorders and suggest that the cerebellum may play a more prominent role in social cognition than previously appreciated.

## Methods

### Participants

#### 4-Repeat Tauopathies

Data used in the preparation of this manuscript were obtained from the 4-Repeat Neuroimaging Initiative (4RTNI) database (http://4rtni-ftldni.ini.usc.edu/). 4RTNI was launched in early 2011 and is funded through the National Institute of Aging and The Tau Research Consortium. The primary goal of 4RTNI is to identify neuroimaging and biomarker indicators for disease progression in the 4-repeat tauopathy neurodegenerative diseases, progressive supranuclear palsy (PSP) and corticobasal degeneration (CBD). The Principal Investigator of 4RTNI is Dr. Adam Boxer, MD, PhD, at the University of California, San Francisco. Twenty-seven participants with CBS and 31 with PSP were recruited at University of California, San Francisco (UCSF) as part of the 4 Repeat Tauopathy Neuroimaging Initiative (4RTNI) study. The study was approved by the Institutional Review Board of UCSF site and all subjects, or their legal guardians gave informed written consent. Patients with PSP met the National Institute of Neurological Disorders and Stroke/Society for PSP-Richardson syndrome as modified for the AL-108–231 study^49,50^. Participants with CBS met Armstrong criteria for possible or probable CBS-CBD subtype (CBS)^51^. All subjects were evaluated at baseline with clinical rating scales, and MRI scans. The Mini-Mental State Examination (MMSE)^52^, the Unified Parkinson’s Disease Rating Scale (UPDRS), the Progressive Supranuclear Palsy Rating Scale (PSPRS), the Clinical Dementia Rating (CDR) score, the Functional Activity Questionnaire (FAQ), the Schwab and England Activities of Daily Living (SEADL) scale and 15-item Geriatric Depression Scale (GDS-15) were administered. The participants also performed a neuropsychological evaluation, including forward and backward digit span tests from the Wechsler Memory Scale III, the letter fluency and category fluency tests modified trail-making task, the Boston naming test, the California Verbal Learning Test and the modified Rey-Osterrieth copy performance. As measure of empathy, we used the Interpersonal Reactivity Index (IRI), a self-reporting questionnaire consisting of four subscales^53^ previously used for dementia and traumatic brain injury patients^8,54^. Two subscales were used to measure cognitive and emotional aspects of empathy, respectively the Perspective Taking (PT, the tendency to spontaneously imagine the cognitive perspective of another person) and the Empathic Concern (EC, the other-centered emotional response resulting from the perception of another’s emotional state). A total score of empathy was also obtained summing PT and EC subscales.

### Behavioral variant frontotemporal dementia

Data used in the preparation of this retrospective study were obtained from the Frontotemporal Lobar Degeneration Neuroimaging Initiative (FTLDNI) database (for up-to-date information on participation and protocol https://4rtni-ftldni.ini.usc.edu). We considered 15 patients with bvFTD who had valid baseline T1-weighted MR images. To avoid potential bias derived from different imaging protocols, we selected exclusively images acquired at the University of California, San Francisco (UCSF), i.e., the largest recruiting center. All the patients underwent comprehensive neurological, neuropsychological, and functional assessments and were diagnosed according to the current diagnostic criteria^55^.

Subjects with bvFTD that form this cohort received the same clinical assessment than 4-Repeat Tauopathies cohort, including Interpersonal Reactivity Index evaluation.

### MRI acquisition and processing

Structural T1-weighted (T1w) MR images were collected using 3 T Siemens Trio Tim system equipped with a 12-channel head coil. Acquisition parameters of structural MR images, using a three-dimensional magnetization-prepared rapid gradient echo (3D-MPRAGE) scheme, were TR/TE/TI = 2300/3/900 ms, flip angle of 9°, sagittal orientation with 256×240×160 matrix size, 1 mm^3^ isotropic voxel resolution. Each MRI volume was visually inspected for gross structural alterations and artifacts. Voxel-based morphometry (VBM) analysis was performed with CAT12 toolbox (Structural Brain Mapping Group, Jena University Hospital, Jena, Germany) implemented in SPM12 (Statistical Parametric Mapping, Institute of Neurology, London, UK). The structural MR imaging data were preprocessed with default settings of the CAT12 toolbox, including corrections for bias-field inhomogeneities, segmentation into gray matter (GM), white matter, and cerebrospinal fluid, followed by spatial normalization to the DARTEL template in MNI space (voxelsize: 1.5 mm×1.5 mm×1.5 mm). Normalized images were modulated to guarantee that relative volumes were preserved following the spatial normalization procedure. Next, the preprocessed GM data were smoothed with an 8 mm full-width-half-maximum (FWHM) isotropic Gaussian kernel. An optimal GM mask was also generated from all normalized images using the SPM12 Masking toolbox and the Luo–Nichols anti-mode method of automatic thresholding^56^.

### Statistical analysis

Demographic, clinical and neuropsychological data analysis was performed with IBM SPSS Statistics 23 (IBM Corporation, New York, EUA). Normality of the data was tested using the Shapiro–Wilk test, followed by ANOVA or Kruskal-Wallis test, and Benjamini-Hochberg correction for multiple comparison, accordingly. Categorical variables were compared with Chi-squared test. The associations between empathy score and motor and clinical variables were explored using Spearman’s correlations, corrected for age, sex, education and disease duration. Statistical significance was considered when *p*-value < 0.003, after Bonferroni correction for multiple comparisons.

Whole-brain multiple-regression analyses were computed by using modulated GM images as dependent variables, the IRI total scores and IRI subscores as regressors. Age and sex were used as covariate, together with the total intracranial volume to correct for individual differences in head size and CDR Sum of Boxes to control for disease severity. Statistical analyses were performed using the Threshold-Free Cluster Enhancement (TFCE) from *FSL-randomise*, a nonparametric permutation-based approach performed with 5000 permutations^57^ Only clusters with *p* < 0.05 after correcting for family-wise error (FWE) were considered statistically significant.

### Reporting summary

Further information on research design is available in the Nature Research Reporting Summary linked to this article.

### Supplementary information

Supplementary tables

reporting summary

## Data Availability

Data used in the preparation of this manuscript were obtained from the 4-Repeat Neuroimaging Initiative (4RTNI) database and the Frontotemporal Lobar Degeneration Neuroimaging Initiative (FTLDNI) (http://4rtni-ftldni.ini.usc.edu/). 4RTNI was launched in early 2011 and is funded through the National Institute of Aging and The Tau Research Consortium. The primary goal of 4RTNI is to identify neuroimaging and biomarker indicators for disease progression in the 4-repeat tauopathy neurodegenerative diseases, progressive supranuclear palsy (PSP) and corticobasal degeneration (CBD). FTLDNI is also founded through the National Institute of Aging, and started in 2010. The primary goals of FTLDNI are to identify neuroimaging modalities and methods of analysis for tracking frontotemporal lobar degeneration (FTLD) and to assess the value of imaging versus other biomarkers in diagnostic roles. The Principal Investigator of 4RTNI is Dr. Adam Boxer, MD, PhD, at the University of California, San Francisco. The data is the result of collaborative efforts at four sites in North America. For more information on 4RTNI, please visit: http://memory.ucsf.edu/research/studies/4rtni.

## Code availability

The underlying code for this study is not publicly available but may be made available to qualified researchers on reasonable request from the corresponding author.
