# Supplementary material for: Grey-matter correlates of empathy in 4-Repeat Tauopathies
Source: NPJ Parkinsons Dis. 2023 Sep 27;9:138. doi: 10.1038/s41531-023-00576-z (PMC10533505; doi:10.1038/s41531-023-00576-z)
Supplement: Supplementary file 1 — Supplementary tables [file 41531_2023_576_MOESM1_ESM.docx]

|  | **PSP** | | **CBS** | |
| --- | --- | --- | --- | --- |
|  | **p-value** | **R** | **p-value** | **R** |
| PSPRS | 0,123 | -0,377 | 0,133 | -0,368 |
| SEADL | 0,608 | 0,134 | 0,012 | 0,577 |
| UPDRS III | 0,704 | 0,088 | 0,862 | 0,039 |
| Californian Verbal Learning Test | 0,901 | -0,028 | 0,577 | 0,133 |
| modified Trail Making task | 0,564 | -0,134 | 0,182 | 0,340 |
| Digit span: forward | 0,353 | 0,214 | 0,948 | -0,015 |
| Digit span: backword | 0,581 | -0,128 | 0,921 | 0,023 |
| Verbal fluency: D word | 0,599 | 0,119 | 0,140 | 0,333 |
| Verbal fluency: Animal | 0,784 | -0,064 | 0,371 | 0,206 |
| Boston Naming Test | 0,156 | -0,329 | 0,420 | 0,186 |
| GDS 15 scale | 0,198 | -0,285 | 0,177 | -0,344 |
| FAQ | 0,147 | -0,337 | 0,025 | -0,488 |
| CDR Sum of boxes | 0,021 | -0,489 | 0,004 | -0,583 |

**Supplementary material**

*Supplementary Table 1.* Spearman's correlations of total IRI with motor and cognitive scores, corrected for age, gender, education and disease duration. Statistical significance was considered when p < 0.003 (Bonferroni-corrected).

*Supplementary Table 2. Clusters of correlation.*

| **Group** | **Anatomic region** | **Cluster size** | **x** | **y** | **z** | **p-value** |
| --- | --- | --- | --- | --- | --- | --- |
| **PSP** | **Total IRI (sum of EC + PT)** |  |  |  |  | **FWE-corr** |
|  | Cluster 1 | 3002 | -61.5 | -34.5 | -4.5 | 0.02 |
|  | L Mid temporal | 2824 |  |  |  |  |
|  | Cluster 2 | 815 | -52.5 | 33 | 0 | 0.03 |
|  | L frontal inf tri | 555 |  |  |  |  |
|  | L frontal inf orb | 259 |  |  |  |  |
|  | **EC subscore** |  |  |  |  | **FWE-corr** |
|  | Cluster 1 | 2061 | -45 | 25.5 | -3 | 0.03 |
|  | L frontal inf tri | 1011 |  |  |  |  |
|  | L frontal inf orb | 605 |  |  |  |  |
|  | L frontal mid | 292 |  |  |  |  |
|  | Cluster 2 | 1968 | -60 | -34.5 | -6 | 0.04 |
|  | L Mid temporal | 1912 |  |  |  |  |
|  | L sup temporal | 53 |  |  |  |  |
|  | Cluster 3 | 1312 | -48 | -42 | 39 | 0.02 |
|  | L Parietal Inf | 1154 |  |  |  |  |
|  | L Supramarginal | 101 |  |  |  |  |
|  | Cluster 4 | 1070 | 6 | -85.5 | -39 | 0.04 |
|  | R cerebellum crus 2 | 823 |  |  |  |  |
|  | R cerebellum 7b | 70 |  |  |  |  |
|  | R cerebellum 8 | 67 |  |  |  |  |
|  | **PT subscore** |  |  |  |  | **FWE-corr** |
|  | Cluster 1 | 1624 | -61.5 | -34.5 | -4.5 | 0.03 |
|  | L Temporal Mid | 1532 |  |  |  |  |
| **CBS** | **Total IRI (sum of EC + PT)** |  |  |  |  | **FWE-corr** |
|  | Cluster 1 | 1652 | -3 | 3 | -12 | 0.04 |
|  | L temporal pole sup | 553 |  |  |  |  |
|  | L temporal pole mid | 168 |  |  |  |  |
|  | L parahippocampal | 152 |  |  |  |  |
|  | L Amygdala | 138 |  |  |  |  |
|  | L hippocampus | 63 |  |  |  |  |
|  | **EC subscore** |  |  |  |  | **FWE-corr** |
|  | Cluster 1 | 18817 | -36 | 9 | -30 | 0.01 |
|  | L temporal pole mid | 905 |  |  |  |  |
|  | R temporal pole mid | 1011 |  |  |  |  |
|  | L temporal pole sup | 1985 |  |  |  |  |
|  | L parahippocampal | 1471 |  |  |  |  |
|  | L hippocampus | 1356 |  |  |  |  |
|  | R hippocampus | 700 |  |  |  |  |
|  | L amygdala | 508 |  |  |  |  |
|  | L putamen | 502 |  |  |  |  |
|  | Cluster 2 | 5131 | 0 | -69 | -39 | 0.03 |
|  | R cerebellum 9 | 734 |  |  |  |  |
|  | L cerebellum 9 | 669 |  |  |  |  |
|  | R cerebellum 8 | 541 |  |  |  |  |
|  | L cerebellum 8 | 505 |  |  |  |  |
|  | Vermis 8 | 430 |  |  |  |  |
|  | Vermis 9 | 357 |  |  |  |  |
|  | Cluster 3 | 3808 | 36 | -42 | -39 | 0.03 |
|  | R cerebellum 6 | 1198 |  |  |  |  |
|  | R cerebellum crus1 | 1039 |  |  |  |  |
|  | R cerebellum crus2 | 581 |  |  |  |  |
|  | **PT subscore** |  |  |  |  | **p-uncorr** |
|  | Cluster 1 | 689 | -3 | 4.5 | -12 | 0.001 |
|  | none | 655 |  |  |  |  |
|  | Cluster 2 | 388 | -10.5 | -4.5 | -21 | 0.003 |
|  | L amygdala | 17 |  |  |  |  |
|  | L Parahippocampal | 69 |  |  |  |  |
|  | L Hippocampus | 144 |  |  |  |  |
|  | Cluster 3 | 312 | 37.5 | -40.5 | -42 | 0.003 |
|  | R Cerebellum crus2 | 79 |  |  |  |  |
|  | R cerebellum 7b | 94 |  |  |  |  |
|  | R cerebellum 8 | 77 |  |  |  |  |
| **bvFTD** | **Total IRI (sum of EC + PT)** |  |  |  |  | **p-uncorr** |
|  | Cluster 1 | 232 | -49.5 | -12 | 33 | 0.002 |
|  | L Postcentral | 222 |  |  |  |  |
|  | Cluster 2 | 162 | 22.5 | -22.5 | 61.5 | 0.002 |
|  | R Precentral | 115 |  |  |  |  |
|  | **EC subscore** |  |  |  |  | **p-uncorr** |
|  | Cluster 1 | 237 | -46.5 | -16.5 | 36 | 0.001 |
|  | L Postcentral | 223 |  |  |  |  |
|  | Cluster 2 | 137 | 21 | -24 | 58.5 | 0.03 |
|  | R Precentral | 92 |  |  |  |  |
|  | **PT subscore** |  |  |  |  | **p-uncorr** |
|  | Cluster 1 | 224 | -46.5 | -15 | 34.5 | 0.002 |
|  | L Postcentral | 218 |  |  |  |  |
|  | Cluster 2 | 182 | 24 | -24 | 61.5 | 0.003 |
|  | R Precentral | 131 |  |  |  |  |
