# Supplementary material for: Grey-matter correlates of empathy in 4-Repeat Tauopathies
Source: NPJ Parkinsons Dis. 2023 Sep 27;9:138. doi: 10.1038/s41531-023-00576-z (PMC10533505; doi:10.1038/s41531-023-00576-z)
Supplement: Supplementary file 2 — reporting summary [file 41531_2023_576_MOESM2_ESM.pdf]

nature portfolio

Corresponding author(s): Giancarlo Logroscino

Last updated by author(s): Aug 23, 2023

Reporting Summary

Nature Portfolio wishes to improve the reproducibility of the work that we publish. This form provides structure for consistency and transparency in reporting. For further information on Nature Portfolio policies, see our Editorial Policies and the Editorial Policy Checklist.

Statistics

For all statistical analyses, confirm that the following items are present in the figure legend, table legend, main text, or Methods section.

n/a	Confirmed	
☐	☒	The exact sample size (*n*) for each experimental group/condition, given as a discrete number and unit of measurement
☐	☒	A statement on whether measurements were taken from distinct samples or whether the same sample was measured repeatedly
☐	☒	The statistical test(s) used AND whether they are one- or two-sided<br>*Only common tests should be described solely by name; describe more complex techniques in the Methods section.*
☐	☒	A description of all covariates tested
☐	☒	A description of any assumptions or corrections, such as tests of normality and adjustment for multiple comparisons
☐	☒	A full description of the statistical parameters including central tendency (e.g. means) or other basic estimates (e.g. regression coefficient) AND variation (e.g. standard deviation) or associated estimates of uncertainty (e.g. confidence intervals)
☐	☒	For null hypothesis testing, the test statistic (e.g. *F*, *t*, *r*) with confidence intervals, effect sizes, degrees of freedom and *P* value noted<br>*Give P values as exact values whenever suitable.*
☒	☐	For Bayesian analysis, information on the choice of priors and Markov chain Monte Carlo settings
☒	☐	For hierarchical and complex designs, identification of the appropriate level for tests and full reporting of outcomes
☒	☐	Estimates of effect sizes (e.g. Cohen's *d*, Pearson's *r*), indicating how they were calculated

Our web collection on statistics for biologists contains articles on many of the points above.

Software and code

Policy information about availability of computer code

Data collection	The datasets generated and analysed during the current study are available in the 4-Repeat Tau Neuroimaging Initiative (4RTNI) repository, http://4rtni-ftldni.ini.usc.edu/. Data collection and sharing for this project was funded by the Frontotemporal Lobar Degeneration Neuroimaging Initiative (National Institutes of Health Grant R01 AG032306). The study is coordinated through the University of California, San Francisco, Memory and Aging Center. FTLDNI data are disseminated by the Laboratory for Neuro Imaging at the University of Southern California.
Data analysis	The underlying code for this study is not publicly available but may be made available to qualified researchers on reasonable request from the corresponding author.

For manuscripts utilizing custom algorithms or software that are central to the research but not yet described in published literature, software must be made available to editors and reviewers. We strongly encourage code deposition in a community repository (e.g. GitHub). See the Nature Portfolio guidelines for submitting code & software for further information.

Data

Policy information about availability of data

All manuscripts must include a data availability statement. This statement should provide the following information, where applicable:

- Accession codes, unique identifiers, or web links for publicly available datasets
- A description of any restrictions on data availability
- For clinical datasets or third party data, please ensure that the statement adheres to our policy

The datasets generated and analysed during the current study are available in the 4-Repeat Tau Neuroimaging Initiative (4RTNI) repository, http://4rtni-ftldni.ini.usc.edu/. Data collection and sharing for this project was funded by the Frontotemporal Lobar Degeneration Neuroimaging Initiative (National Institutes of Health Grant R01 AG032306). The study is coordinated through the University of California, San Francisco, Memory and Aging Center. FTLDNI data are disseminated by the Laboratory for Neuro Imaging at the University of Southern California.

Research involving human participants, their data, or biological material

Policy information about studies with human participants or human data. See also policy information about sex, gender (identity/presentation), and sexual orientation and race, ethnicity and racism.

Reporting on sex and gender	We use term sex as biological attribute and self-reported information. In our analysis we used both sex. No information over gender distribution was collected. In results section and Table 1 we reported sex-based statistical analysis.
Reporting on race, ethnicity, or other socially relevant groupings	No information about race or ethnicity was collected.
Population characteristics	All subjects were evaluated at baseline with clinical rating scales, and MRI scans. The Mini-Mental State Examination (MMSE), the Unified Parkinson's Disease Rating Scale (UPDRS), the Progressive Supranuclear Palsy Rating Scale (PSPRS), the Clinical Dementia Rating (CDR) score, the Functional Activity Questionnaire (FAQ), the Schwab and England Activities of Daily Living (SEADL) scale and 15-item Geriatric Depression Scale (GDS-15) were administered. The participants also performed a neuropsychological evaluation, including forward and backward digit span tests from the Wechsler Memory Scale III, the letter fluency and category fluency tests modified trail-making task, the Boston naming test, the California Verbal Learning Test and the modified Rey-Osterrieth copy performance. As measure of empathy, we used the Interpersonal Reactivity Index (IRI), a self-reporting questionnaire consisting of four subscales previously used for dementia and traumatic brain injury patients. Two subscales were used to measure cognitive and emotional aspects of empathy, respectively the Perspective Taking (PT, the tendency to spontaneously imagine the cognitive perspective of another person) and the Empathic Concern (EC, the other-centered emotional response resulting from the perception of another's emotional state). A total score of empathy was also obtained summing PT and EC subscales.
Recruitment	Patients with PSP met the National Institute of Neurological Disorders and Stroke/Society for PSP-Richardson syndrome as modified for the AL-108-231 study. Participants with CBS met Armstrong criteria for possible or probable CBS-CBD subtype (CBS).
Ethics oversight	The study was approved by the Institutional Review Board of UCSF site and all subjects, or their legal guardians gave informed written consent.

Note that full information on the approval of the study protocol must also be provided in the manuscript.

Field-specific reporting

Please select the one below that is the best fit for your research. If you are not sure, read the appropriate sections before making your selection.

☒ Life sciences ☐ Behavioural & social sciences ☐ Ecological, evolutionary & environmental sciences

For a reference copy of the document with all sections, see nature.com/documents/nr-reporting-summary-flat.pdf

Life sciences study design

All studies must disclose on these points even when the disclosure is negative.

Sample size	Our sample size was determined by the rarity of the described pathologies. Only for a rare disease is an underpowered study justified, due to logistics as the data from such a study is helpful in meta-analysis.
Data exclusions	In our study we included patients based on all clinical data and MRI availability.
Replication	Our results are replicable based on all information of age, sex, total intracranial volume, CDR Sum of Boxes and Interpersonal Reactivity Index scores combined with T1 MRI acquisition.
Randomization	in our analysis we used age, sex, total intracranial volume, CDR Sum of Boxes as covariates.

Blinding	Blinding was not relevant to this study because we have performed a correlation analysis

Reporting for specific materials, systems and methods

We require information from authors about some types of materials, experimental systems and methods used in many studies. Here, indicate whether each material, system or method listed is relevant to your study. If you are not sure if a list item applies to your research, read the appropriate section before selecting a response.

Materials & experimental systems

n/a	Involved in the study
☐	☐ Antibodies
☐	☐ Eukaryotic cell lines
☐	☐ Palaeontology and archaeology
☐	☐ Animals and other organisms
☐	☒ Clinical data
☐	☐ Dual use research of concern
☐	☐ Plants

Methods

n/a	Involved in the study
☐	☐ ChIP-seq
☐	☐ Flow cytometry
☐	☒ MRI-based neuroimaging

Antibodies

Antibodies used	*Describe all antibodies used in the study; as applicable, provide supplier name, catalog number, clone name, and lot number.*
Validation	*Describe the validation of each primary antibody for the species and application, noting any validation statements on the manufacturer's website, relevant citations, antibody profiles in online databases, or data provided in the manuscript.*

Eukaryotic cell lines

Policy information about cell lines and Sex and Gender in Research

Cell line source(s)	*State the source of each cell line used and the sex of all primary cell lines and cells derived from human participants or vertebrate models.*
Authentication	*Describe the authentication procedures for each cell line used OR declare that none of the cell lines used were authenticated.*
Mycoplasma contamination	*Confirm that all cell lines tested negative for mycoplasma contamination OR describe the results of the testing for mycoplasma contamination OR declare that the cell lines were not tested for mycoplasma contamination.*
Commonly misidentified lines (See ICLAC register)	*Name any commonly misidentified cell lines used in the study and provide a rationale for their use.*

Palaeontology and Archaeology

Specimen provenance	*Provide provenance information for specimens and describe permits that were obtained for the work (including the name of the issuing authority, the date of issue, and any identifying information). Permits should encompass collection and, where applicable, export.*
Specimen deposition	*Indicate where the specimens have been deposited to permit free access by other researchers.*
Dating methods	*If new dates are provided, describe how they were obtained (e.g. collection, storage, sample pretreatment and measurement), where they were obtained (i.e. lab name), the calibration program and the protocol for quality assurance OR state that no new dates are provided.*

☐ Tick this box to confirm that the raw and calibrated dates are available in the paper or in Supplementary Information.

Ethics oversight	*Identify the organization(s) that approved or provided guidance on the study protocol, OR state that no ethical approval or guidance was required and explain why not.*

Note that full information on the approval of the study protocol must also be provided in the manuscript.

Animals and other research organisms

Policy information about studies involving animals; ARRIVE guidelines recommended for reporting animal research, and Sex and Gender in Research

Laboratory animals	*For laboratory animals, report species, strain and age OR state that the study did not involve laboratory animals.*
Wild animals	*Provide details on animals observed in or captured in the field; report species and age where possible. Describe how animals were caught and transported and what happened to captive animals after the study (if killed, explain why and describe method; if released, say where and when) OR state that the study did not involve wild animals.*
Reporting on sex	*Indicate if findings apply to only one sex; describe whether sex was considered in study design, methods used for assigning sex. Provide data disaggregated for sex where this information has been collected in the source data as appropriate; provide overall numbers in this Reporting Summary. Please state if this information has not been collected. Report sex-based analyses where performed, justify reasons for lack of sex-based analysis.*
Field-collected samples	*For laboratory work with field-collected samples, describe all relevant parameters such as housing, maintenance, temperature, photoperiod and end-of-experiment protocol OR state that the study did not involve samples collected from the field.*
Ethics oversight	*Identify the organization(s) that approved or provided guidance on the study protocol, OR state that no ethical approval or guidance was required and explain why not.*

Note that full information on the approval of the study protocol must also be provided in the manuscript.

Clinical data

Policy information about clinical studies

All manuscripts should comply with the ICMJE guidelines for publication of clinical research and a completed CONSORT checklist must be included with all submissions.

Clinical trial registration	NCT01804452
Study protocol	http://4rtni-ftldni.ini.usc.edu
Data collection	The datasets generated and analysed during the current study are available in the 4-Repeat Tau Neuroimaging Initiative (4RTNI) repository, http://4rtni-ftldni.ini.usc.edu/. Data collection and sharing for this project was funded by the Frontotemporal Lobar Degeneration Neuroimaging Initiative (National Institutes of Health Grant R01 AG032306). The study is coordinated through the University of California, San Francisco, Memory and Aging Center. FTLDNI data are disseminated by the Laboratory for Neuro Imaging at the University of Southern California.
Outcomes	Two subscales were used to measure cognitive and emotional aspects of empathy, respectively the Perspective Taking (PT, the tendency to spontaneously imagine the cognitive perspective of another person) and the Empathic Concern (EC, the other-centered emotional response resulting from the perception of another's emotional state). A total score of empathy was also obtained summing PT and EC subscales.

Dual use research of concern

Policy information about dual use research of concern

Hazards

Could the accidental, deliberate or reckless misuse of agents or technologies generated in the work, or the application of information presented in the manuscript, pose a threat to:

No	Yes	
☐	☐	Public health
☐	☐	National security
☐	☐	Crops and/or livestock
☐	☐	Ecosystems
☐	☐	Any other significant area

Experiments of concern

Does the work involve any of these experiments of concern:

No	Yes	
☐	☐	Demonstrate how to render a vaccine ineffective
☐	☐	Confer resistance to therapeutically useful antibiotics or antiviral agents
☐	☐	Enhance the virulence of a pathogen or render a nonpathogen virulent
☐	☐	Increase transmissibility of a pathogen
☐	☐	Alter the host range of a pathogen
☐	☐	Enable evasion of diagnostic/detection modalities
☐	☐	Enable the weaponization of a biological agent or toxin
☐	☐	Any other potentially harmful combination of experiments and agents

Plants

Seed stocks
Report on the source of all seed stocks or other plant material used. If applicable, state the seed stock centre and catalogue number. If plant specimens were collected from the field, describe the collection location, date and sampling procedures.

Novel plant genotypes
Describe the methods by which all novel plant genotypes were produced. This includes those generated by transgenic approaches, gene editing, chemical/radiation-based mutagenesis and hybridization. For transgenic lines, describe the transformation method, the number of independent lines analyzed and the generation upon which experiments were performed. For gene-edited lines, describe the editor used, the endogenous sequence targeted for editing, the targeting guide RNA sequence (if applicable) and how the editor was applied.

Authentication
Describe any authentication procedures for each seed stock used or novel genotype generated. Describe any experiments used to assess the effect of a mutation and, where applicable, how potential secondary effects (e.g. second site T-DNA insertions, mosiacism, off-target gene editing) were examined.

ChIP-seq

Data deposition

☐ Confirm that both raw and final processed data have been deposited in a public database such as GEO.

☐ Confirm that you have deposited or provided access to graph files (e.g. BED files) for the called peaks.

Data access links
May remain private before publication.
For "Initial submission" or "Revised version" documents, provide reviewer access links. For your "Final submission" document, provide a link to the deposited data.

Files in database submission
Provide a list of all files available in the database submission.

Genome browser session
(e.g. UCSC)
Provide a link to an anonymized genome browser session for "Initial submission" and "Revised version" documents only, to enable peer review. Write "no longer applicable" for "Final submission" documents.

Methodology

Replicates
Describe the experimental replicates, specifying number, type and replicate agreement.

Sequencing depth
Describe the sequencing depth for each experiment, providing the total number of reads, uniquely mapped reads, length of reads and whether they were paired- or single-end.

Antibodies
Describe the antibodies used for the ChIP-seq experiments; as applicable, provide supplier name, catalog number, clone name, and lot number.

Peak calling parameters
Specify the command line program and parameters used for read mapping and peak calling, including the ChIP, control and index files used.

Data quality
Describe the methods used to ensure data quality in full detail, including how many peaks are at FDR 5% and above 5-fold enrichment.

Software
Describe the software used to collect and analyze the ChIP-seq data. For custom code that has been deposited into a community repository, provide accession details.

Flow Cytometry

Plots

Confirm that:

☐ The axis labels state the marker and fluorochrome used (e.g. CD4-FITC).

☐ The axis scales are clearly visible. Include numbers along axes only for bottom left plot of group (a 'group' is an analysis of identical markers).

☐ All plots are contour plots with outliers or pseudocolor plots.

☐ A numerical value for number of cells or percentage (with statistics) is provided.

Methodology

Sample preparation	*Describe the sample preparation, detailing the biological source of the cells and any tissue processing steps used.*
Instrument	*Identify the instrument used for data collection, specifying make and model number.*
Software	*Describe the software used to collect and analyze the flow cytometry data. For custom code that has been deposited into a community repository, provide accession details.*
Cell population abundance	*Describe the abundance of the relevant cell populations within post-sort fractions, providing details on the purity of the samples and how it was determined.*
Gating strategy	*Describe the gating strategy used for all relevant experiments, specifying the preliminary FSC/SSC gates of the starting cell population, indicating where boundaries between "positive" and "negative" staining cell populations are defined.*

☐ Tick this box to confirm that a figure exemplifying the gating strategy is provided in the Supplementary Information.

Magnetic resonance imaging

Experimental design

Design type	Structural T1 weighted MRI acquisition
Design specifications	Our experiment consists of a single MRI acquisition at baseline
Behavioral performance measures	n/a

Acquisition

Imaging type(s)	Structural MRI
Field strength	3 tesla
Sequence & imaging parameters	Acquisition parameters of structural MR images, using a three-dimensional magnetization-prepared rapid gradient echo (3D-MPRAGE) scheme, were TR/TE/TI = 2300/3/900 ms, flip angle of 9°, sagittal orientation with 256×240×160 matrix size, 1 mm3 isotropic voxel resolution.
Area of acquisition	Whole brain scan

Diffusion MRI ☐ Used ☒ Not used

Preprocessing

Preprocessing software	CAT12 toolbox (Structural Brain Mapping Group, Jena University Hospital, Jena, Germany) implemented in SPM12 (Statistical Parametric Mapping, Institute of Neurology, London, UK)
Normalization	The structural MR imaging data were non-linear normalized.
Normalization template	DARTEL template in MNI space (voxelsize: 1.5 mm x 1.5 mm x 1.5 mm)
Noise and artifact removal	The structural MR imaging data were preprocessed with default settings of the CAT12 toolbox, including corrections for bias-field inhomogeneities, segmentation into gray matter (GM), white matter, and cerebrospinal fluid, followed by spatial normalization to the DARTEL template in MNI space (voxelsize: 1.5 mm x 1.5 mm x 1.5 mm). Normalized images were modulated to guarantee that relative volumes were preserved following the spatial normalization procedure. Next, the preprocessed GM data were smoothed with an 8mm full-width-half-maximum (FWHM) isotropic Gaussian kernel. An optimal GM mask was also generated from all normalized images using the SPM12 Masking toolbox and the Luo–Nichols anti-mode

method of automatic thresholding.

Volume censoring

n/a

Statistical modeling & inference

Model type and settings

Voxel-based morphometry (VBM) analysis was performed with CAT12 toolbox (Structural Brain Mapping Group, Jena University Hospital, Jena, Germany) implemented in SPM12 (Statistical Parametric Mapping, Institute of Neurology, London, UK).

Effect(s) tested

Whole-brain multiple-regression analyses were computed by using modulated GM images as dependent variables, the IRI total scores and IRI subscores as regressors. Age and sex were used as covariate, together with the total intracranial volume to correct for individual differences in head size and CDR Sum of Boxes to control for disease severity.

Specify type of analysis: ☒ Whole brain ☐ ROI-based ☐ Both

Statistic type for inference

(See Eklund et al. 2016)

Voxel-wise analysis

Correction

Statistical analyses were performed using the Threshold-Free Cluster Enhancement (TFCE) from FSL-randomise, a nonparametric permutation-based approach performed with 5000 permutations57 Only clusters with $p < 0.05$ after correcting for family-wise error (FWE) were considered statistically significant.

Models & analysis

n/a	Involved in the study
☒	☐ Functional and/or effective connectivity
☒	☐ Graph analysis
☒	☐ Multivariate modeling or predictive analysis
